# Consumption and Sources of Dietary Salt in Family Members in Beijing

**DOI:** 10.3390/nu7042719

**Published:** 2015-04-10

**Authors:** Fang Zhao, Puhong Zhang, Lu Zhang, Wenyi Niu, Jianmei Gao, Lixin Lu, Caixia liu, Xian Gao

**Affiliations:** 1 The George Institute for Global Health at Peking University Health Science Center, Level 18, Tower B, Horizon Tower, 6, Zhichun Road, Haidian District, Beijing 100088, China; E-Mail: fzhao@georgeinstitute.org.cn; 2 Chinese Center for Disease Control and Prevention, 155, Changbai Road, Changping District, Beijing 102206, China; 3 Center for Health Policy and Management, Institute of Medical Information, Chinese Academy of Medical Sciences, 3, Yabao Road, Chaoyang District, Beijing 100020, China; E-Mail: zhang.lu@imicams.ac.cn; 4 Department of Social Medicine and Health education, School of Public Health, Peking University, 38 Xueyuan Road, Haidian District, Beijing 100191, China; E-Mail: health1956@163.com; 5 Huairou Center for Disease Control and Prevention, 23, Fule North Street, Huairou District, Beijing 101400, China; E-Mails: hrgaojm@sina.com (J.G.); hrcdcmb@yeah.net (C.L.); 6 Xicheng Center for Disease Control and Prevention, 38, Deshengmenwai Street, Xicheng District, Beijing 100011, China; E-Mails: lulixin518@sina.com (L.L.); xichengcdc@126.com (X.G.)

**Keywords:** salt intake, salt sources

## Abstract

In China, few people are aware of the amount and source of their salt intake. We conducted a survey to investigate the consumption and sources of dietary salt using the “one-week salt estimation method” by weighing cooking salt and major salt-containing food, and estimating salt intake during dining out based on established evidence. Nine hundred and three families (1981 adults and 971 children) with students in eight primary or junior high schools in urban and suburban Beijing were recruited. On average, the daily dietary salt intake of family members in Beijing was 11.0 (standard deviation: 6.2) g for children and adolescents (under 18 years old), 15.2 (9.1) g for adults (18 to 59 years old), and 10.2 (4.8) g for senior citizens (60 years old and over), respectively. Overall, 60.5% of dietary salt was consumed at home, and 39.5% consumed outside the home. Approximately 90% of the salt intake came from cooking (household cooking and cafeteria or restaurant cooking), while less than 10% came from processed food. In conclusion, the dietary salt intake in Beijing families far surpassed the recommended amounts by World Health Organization, with both household cooking and dining-out as main sources of salt consumption. More targeted interventions, especially education about major sources of salt and corresponding methods for salt reduction should be taken to reduce the risks associated with a high salt diet.

## 1. Introduction

Convincing evidence has shown that high sodium intake is associated with an increased risk of high blood pressure, which is a major risk factor for coronary heart disease and both forms of stroke (ischemic and hemorrhagic) [1]. The joint World Health Organization (WHO)/the Food and Agriculture Organization (FAO) report on diet, nutrition, and the prevention of chronic diseases (WHO, 2003) stated that the population nutrient intake goal for salt should be <5 g/day [1]; the Chinese Nutrition Society recommends a daily salt intake of less than 6 g for Chinese adults [2]. However, the results of the China National Nutrition and Health Survey, a large cross-sectional study carried out in 2002 with a nationally representative sample of the whole population using dietary survey, showed that the average salt intake of Chinese residents was 12 g per standard person per day [3], much higher than the recommended amount.

To reduce salt intake, WHO issued the following recommendations: (1) estimate baseline salt intake; (2) identify the major sources of salt consumption; and (3) propose measures for population salt reduction. In industrialized countries, about 75% of sodium consumed comes from processed foods and food eaten away from home [4]. In China, the salt added in cooking and in sauces and seasonings represents 82.8% of sodium in the diet [5]. Few people, however, are aware of the amount or the sources of their salt intake, the amount of salt reduction needed and the most effective ways for reducing salt intake by themselves. The lack of awareness of the amount and sources of salt consumption is a key obstacle in salt reduction because dietary sources of salt must be identified and evaluated to effectively implement population-based salt reduction recommendations [5].

Several methods can be used to estimate salt intake: 24-h urine collection, duplicate diets and dietary surveys [4]. Twenty-four-hour urine collection, while accurately estimating salt intake and considered the “gold standard,” cannot distinguish between sources of salt. Furthermore, collecting 24-h urine samples from a representative sample of the population is challenging and resource-intensive [6]. Duplicate diets are complicated to implement, and dietary surveys have proved to be inaccurate sometimes even showing poor correlation with results from urine collection [7]. Therefore, to estimate dietary salt intake and identify the sources of salt intake in family members in Beijing, China, we used a simplified one-week dietary record method named the “one week estimation method”, which combined weighing cooking salt and major salt containing foods with evidence based estimation of salt consumption when dining out.

## 2. Methods

### 2.1. Participants

Study participants were recruited from families in urban (Xicheng District) and suburban (Huairou District) Beijing in 2011. To increase compliance and facilitate project management, we recruited study families through public primary and junior high schools. In total, we enrolled eight schools, 2 primary and 2 junior high schools from urban Beijing, and 2 primary and 2 junior high schools from suburban Beijing. Eligible families were those with a child from the enrolled schools in grades 3–5 (primary schools) or grades 7–8 (junior high schools) who were willing to participate in the study. As participating students would be tasked with recording their family’s diet, we excluded students in grades 1–2 (too young to understand) and students in grade 6 and 9 (too busy with school examinations). There were several reasons for choosing students to be the key persons for this study: (1) In China, most students take homework (such as recording their family’s diet) given by their teacher very seriously resulting in higher compliance rates than those of adult family members; (2) primary and junior high school students usually live with their families and are familiar with the daily diets of each family member; (3) the information needed was simple for these students to accurately collect, and, if necessary, they could get assistance from their trained parents who had been taught to how to use the family kitchen scale appropriately and record the dietary information correctly.

The students collected information regarding the eating habits of each family member during the week, including the number of meals consumed each day, where the food was consumed, how many guests ate with the family each day and the proportion of food that was usually discarded. We provided each family with an electric kitchen scale to weigh salt and other foods that they were trained to use during health education classes. We obtained informed consent from each family through the participating students.

The research project was approved by Chinese Center for Disease Control and Prevention (No. 201515).

### 2.2. Salt Intake Evaluation Method

A simplified one-week salt estimation method named the “one week salt estimation method” was designed to measure each family member’s daily salt intake and determine the sources of salt in the diet. The methodology of this new method was published previously [8]. In brief, this method estimates salt intake from three sources: household cooking, processed food, and cafeterias or restaurants. We recorded cooking salt (including traditional iodized salt, low sodium iodized salt, and other salt) and other salt-containing condiments (including monosodium glutamate, soy sauce, vinegar, and sauces) used in household cooking by weighing all the bottles and salt containers in the household at the start and end of the week. This provided the total salt consumption for the whole household, which was then assigned to individuals. We classified processed food into the six most commonly consumed categories of food (instant noodles; deep-fried dough sticks and cake; steam stuffed bun, dumplings, baked pancake, and bread; processed meats; salted vegetables; and others). Participants recorded the consumed amounts for each category in a pre-printed booklet at the time of consumption. An arithmetic average sodium content for each of the 6 categories of processed food was used to calculate the salt intake and combining with the recorded consumption according to China Food Composition data [9]. Combining the average sodium concentration with the recorded consumption amount of each category, we estimated the sodium intake from processed food. However, considering that most Chinese people understand salt much more easily than sodium, we converted all the sodium results by multiplying 2.54 into salt (sodium chloride) in this article. We calculated the energy intake of each family member from their age, body weight and gender using the Recommended Nutrient Intake (RNIs) of energy in the China dietary nutrition survey reference intake scale [10], and then used the calculated energy intake to allocate the total household salt intake to each household member.The salt consumed from dining out at cafeterias or restaurants was estimated using a previous study by weighing cooking salt and salt-containing condiments that showed in China, salt consumed from dining out was about 1.5 times the salt consumed through household cooking [11,12].

The results from the methodology study [8] showed that the one-week salt estimation method will underestimate salt intake by 1.8 g/person/day with comparison to the 24-h urine collection method. For children, the underestimation was 2.0 g/person/day while for adults it was 1.7 g/person/day. In order to avoid the underestimation, the amount of dietary salt intake was adjusted by different age groups.

### 2.3. Data Collection

We used a two-section questionnaire to collect relevant data on the entire family (rather than on an individual basis). The first section, “Basic Information,” consisted of each family member’s gender, weight and physical activity level. The second section, “One Week Salt Intake Recording Form”, recorded for a one-week period: (1) At a household level, the weight of cooking salt, salt-containing condiments, and fast food/snacks consumed using an SF-400 kitchen scale with an accuracy within 1 g and measurement range of 1–5000 g, the number of guest diners and the proportion of food discarded; (2) at an individual level, whether and where each family member eat their meals.

Both parents and students were informed of the study content and instructed by trained school teachers to use the kitchen scale appropriately and record the dietary information correctly. In general, the students were assigned to weigh the food and perform the dietary recording, asking their parents for help if needed. Parents completed the “Basic Information” section of the questionnaire, and students were instructed to bring the “One Week Salt Intake Recording Form” booklet to school every day for trained teachers to check. Researchers reviewed all completed questionnaires. Incomplete questionnaires were excluded.

### 2.4. Data Analysis

Continuous variables were described as mean (standard deviation) while categorical variables were presented by proportions. To compare differences among groups of area, gender and age for continuous variables, we used generalized estimating equation models to account for clustering within same families. Age was classified into 3 categories: (1) children and adolescents (1 to 17 years old); (2) adults (18 to 59 years old); and (3) senior citizens (60 to 85 years old). Differences were considered statistically significant at *p* < 0.05. All statistical analyses were carried out using SAS 9.3 (SAS Institute Inc., Cary, NC, USA).

## 3. Results

### 3.1. Characteristics of the Participants

One thousand and thirty-four families participated in the study. We collected 910 (88%) questionnaires (one from each family, including information on all family members), and 903 (87.3%) of them were deemed eligible for use in the data analysis. Of the 903 families totaling 2952 participants, 345 (38.2%) were from urban areas, 558 (61.8%) were from rural areas, 562 (61.8%) were primary school student families, and 371 (37.8%) were middle school student families. Among all the family adults who answered the questionnaire, 27.2% (urban: 46.7%; suburban: 15.2%) had college degree or above. A total of 14.2% (urban: 25.5%; suburban: 7.2%) of the mean annual household income in 2010 was over 100,000 RMB. Basic characteristics of all participants are shown in Table 1.

**Table 1:** Basic characteristics of participants.

	Children and Adolescents (1–17 years old)	Adults (18–59 years old)	Senior Citizens (60–85 years old)
Number (*n*)	971	1730	251
Male (%)	47.9	46.6	45.4
Age (x (sd), years)	12.0 (2.5)	39.3 (5.0)	68.3 (5.9)
Weight (x (sd, Kg))	44.9 (15.5)	66.3 (12.8)	63.4 (9.1)
Labor intensity (%)			
Mild	64.5	60.5	80.5
Moderate	33.5	36.0	18.7
Severe	2.1	3.6	0.8
Percentage of meals eaten outside the home (%)			
breakfast	23.2 (33.7)	28.9 (36.4)	4.7 (13.6)
lunch	61.9 (37.3)	54.8 (38.1)	9.7 (19.9)
supper	10.7 (17.7)	18.4 (24.6)	5.1 (13.3)
Area (%)			
urban	37.2	37.2	54.6
suburban	62.8	62.8	45.4

### 3.2. The Amount of Dietary Salt Intake

**Figure 1:**
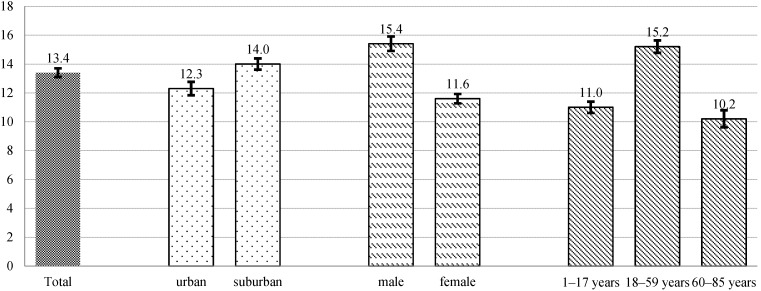
Dietary salt intake by region, gender and age groups (mean, g/person/day). Note: Vertical lines denote ±95% Confidence Interval. There were significant differences in the dietary salt intake between urban and suburban areas (*p* < 0.01), male and female (*p* < 0.01), as well as age groups (*p* < 0.01).

The daily dietary salt intake of all participants was 13.4 (8.3) g/person. There was significant difference in the dietary salt intake between suburban and urban areas. Suburban residents consumed more dietary salt (14.0 (8.4) g/person/day) than urban residents (12.3 (8.0) g/person/day) (*p* < 0.01). Comparing gender differences, men consumed more dietary salt (15.4 (9.5) g/person/day) than women (11.6 (6.6) g/person/day) (*p* < 0.01). There were also significant differences between age groups (*p* < 0.01). Adults consumed more dietary salt (15.2 (9.1) g/person/day) than children and adolescents (11.0 (6.2) g/person/day) and senior citizens (10.2 (4.8) g/person/day) (Figure 1).

### 3.3. Sources of Dietary Salt Intake

Table 2 illustrates the sources of dietary salt intake. Overall, 60.5% (8.1 (4.5) g/person/day) of dietary salt was consumed at home, and 39.5% (5.3 (6.7) g/person/day) consumed out at cafeterias or restaurants. 93.1% of the salt came from cooking (household cooking and cafeteria or restaurant cooking) while 6.9% came from processed food. Compared to their suburban counterparts, urban residents consumed more dietary salt from cafeterias/restaurants and consumed less dietary salt from cooking salt and salt-containing condiments used in household cooking (*p* < 0.01). Men consumed more dietary salt from cafeterias/restaurants but less from all other sources (*p* < 0.01) compared to women. Between age groups, the differences in dietary salt sources were significant (*p* < 0.01). Individuals younger than 17 and between 18 and 59 years old consumed about 40% dietary salt dining out at cafeterias or restaurants while senior citizens consumed less than 10% dining out.

**Table 2:** Sources of dietary salt intake (mean (standard deviation)).

	*N*	Proportion of Different Sources of Dietary Salt (%)				
		At Home				Out
		Subtotal	Cooking Salt	Condiments	Fast Food or Snacks	(At Cafeterias or Restaurants)
Total	2952	60.5 (26.9)	43.7 (23.5)	9.8 (9.8)	6.9 (10.1)	39.5 (26.9)
Area						
urban	1142	56.8 (24.6) **	40.6 (21.7) **	8.9 (10.0) **	7.4 (10.4)	43.1 (24.6) **
suburban	1810	62.8 (28.0)	45.7 (24.4)	10.4 (9.6)	6.6 (9.9)	37.2 (28.0)
Gender						
male	1385	55.7 (27.1) **	40.4 (23.4) **	9.0 (8.9) **	6.4 (9.4) **	44.3 (27.1) **
female	1567	64.7(26.0)	46.7 (23.2)	10.6 (10.4)	7.4 (10.6)	35.4 (26.0)
Age(years)						
1–17	971	59.3 ± 22.2 **	42.7 (20.3) **	9.6 (9.1) **	7.1 (10.0) **	40.7 (22.2) **
18–59	1730	56.7 ± 27.7 **	40.8 (23.7) **	9.3 (9.6) **	6.6 (9.8) *	43.3 (27.7) **
60–85	251	91.1 ± 15.9	67.9 (20.0)	14.4 (12.4)	8.7 (11.8)	8.9 (15.9)

**: *p* < 0.01; *: *p* < 0.05.

**Figure 2:**
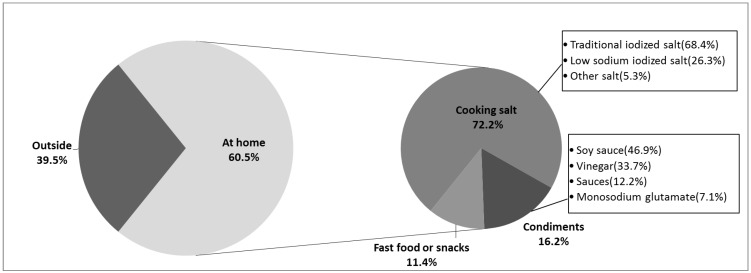
Sources of dietary salt intake.

Of the salt intake consumed at home (6.3 (4.5) g/person/day), the average amount of salt added during cooking was 5.5 (4.1) g/person/day. Cooking salt was the main home salt source accounting for 81.7% (72.2%/(72.2% + 16.2%)) of the total amount consumed in household cooking. The proportion of each source is detailed in Figure 2.

## 4. Discussion

Our study shows that dietary salt intake in family members in Beijing far surpasses the recommended amount by WHO [1]. Overall, 60.5% of dietary salt was consumed at home, and 39.5% consumed out at cafeterias or restaurants. Cooking is still the major source of salt in the diet, making up around 90% of the total salt intake (53.5% from household cooking and 39.5% from cafeteria or restaurant cooking). Our current analysis has two strengths: (1) we estimated salt intake using a combined family based method of dietary survey and weighing major sodium-containing food which has the ability to characterize usual intake based on an individual or household, and is highly correlated with the 24-h urine collection gold standard; (2) both consumption and sources of dietary salt intake were reported.

The INTERMAP Study, an international, cross-sectional, epidemiologic study of 4680 individuals, aged 40 to 59 years, from four countries, showed a mean dietary salt intake of 13.3 ± 5.9 g/person/day among 839 samples from rural China using the 24-h urine collection method [5]. Our result is a little higher. This may be due to a number of factors: (1) Although the INTERMAP Study used the “gold standard” to assess dietary salt intake, that is, sodium excretion from 24-h urine collections which can capture 85% to 90% of the ingested sodium [4], the 24-h urine collection misses approximately 10% of sodium intake excreted in sweat and feces [11]; (2) The participants differ in age and area between the two studies. The participants in the INTERMAP Study were all middle-aged and from rural China. Our study was conducted only in Beijing and included both urban and suburban participants covering a wider age range.

The SMASH study, which conducted in 2011 using three-day dietary recalls and weighing methods recruited 2140 participants between 18 and 69 years old in Shandong province, located in North China, showed an individual dietary salt intake of 14.6 g/day, with 15.0 g/day and 13.6 g/day for rural residents and urban residents respectively [13]. The amount of salt consumption for adults aged 18 to 59 years in our study was 15.2g/person/day. Considering 13.8% of participants in SMASH study were 60 to 69 years old and these people consumed less salt, we conclude the results of our study and those of the 2011 study to be similar. Another study specifically surveyed the Xicheng District (our urban district) and found a much lower mean dietary salt intake of 7.71g/person/day [14]. Our study found the mean to be 12.3 ± 8.0 g/person/day. The discrepancy was found to be a result of weighing only salt, condiments and sauces, but not considering dining out and purchased food eaten at home in the Xicheng study.

Our subgroup analyses showed dietary salt intake in men was significantly higher than that of women. This may be due to men eating a higher quantity of food than women. The dietary salt intake of adults aged 18–59 years old was the highest among all of the age groups. In addition to the higher energy consumption, this may due to their higher percentage of dining out. Previous research [15] conducted in China has indicated that when dining out, dietary structure vastly differs from eating at home. Dining out may result in a lower carbohydrate, animal product-heavy, higher energy/fat, and higher salt meal. Urban residents consumed less dietary salt than people who lived in suburban. This may be a result of improved socio-economic status, and awareness of and accessibility to health related information. In general, urban residents favored less salty foods [16].

This current study analyzed the sources of dietary salt intake for residents in Beijing. These results differ from the INTERMAP Study results as mentioned above. The INTERMAP Study found that most (75.8%) salt was added in home cooking. Other sources included soy sauce (6.4%), noodles and breads (3.8%), pickled vegetables (3.6%), salted eggs (0.8%), and monosodium glutamate (0.6%) [5]. Salt from cafeterias or restaurants, along with purchased fast food or snacks eating at home, was not considered in the INTERMAP Study. This may have been due to changing eating habits and that their participants were from rural China in the 1990s when people seldom had meals away from home, especially in rural areas. With social development, living standards have improved, the pace of living has accelerated and more and more people choose to dine out [17]. As shown in our results, the proportion of salt from cafeterias or restaurants and purchased fast food or snacks eaten at home surpasses 40%. Among those aged 59 or younger and urban residents the proportion is even higher.

According to our results, cooking salt is still the major source of salt intake in household cooking, but the proportion of salt from condiments has increased by 1.8% from data from the China National Nutrition and Health Survey in 2002 [16]. In addition, we found that low sodium salt accounted for around one-fifth (21.5%) of the cooked food, sauces, and condiments consumed within the household, excluding fast food and snacks. This change may be policy-related as the Chinese government began promoting the consumption of low sodium salt in 2011. The government not only promoted the use of low sodium salt in restaurants, cafeterias and in homes, but also carried out health education campaigns in order to raise public awareness [18].

Our study shows that cooking, household cooking and dining out, is still the main source of salt consumption in China suggesting that follow-up interventions can target both modes. We have several suggestions for ways to move forward. For salt reduction in household cooking: (1) Use salt restriction spoons. Previous studies have showed that the correct use of a salt restriction spoon can achieve the aim of limiting salt intake, and it can be used for a wide range of simple, established salt reduction measures for residents during daily life [19]; (2) low sodium salt can be vigorously promoted in the appropriate population. Related studies [20,21] have shown that low sodium salt has had an obvious effect in lowing blood pressure levels. A large-scale, blinded randomized trial aimed to test the acceptability of low sodium salt (65% sodium chloride, 25% potassium chloride, and 10% magnesium sulphate) in rural Northern China showed that people randomized into groups found no marked difference between the saltiness, flavor and overall acceptability of foods cooked with low-sodium salt and normal salt (all *p* > 0.08) [22]. Our results show low sodium salt contributes about 20% of the total salt from household cooking. It is clear that many families have begun to incorporate low sodium salt in their household cooking, so further measures should be taken to increase publicity and promote its usage; (3) as well as to reduce the usage of salt-containing condiments during cooking. Although cooking salt accounted for most of the sodium added through cooking, the proportion from condiments has increased. We strongly recommend a strong public education program since many people may not know they are consuming more than the recommended daily salt intake because of condiments; (4) in Western countries, most salt consumption comes from processed foods and food eaten outside of the home [4]. In China, salt intake largely depends on the family, so the establishment and promotion of salt intake assessment methods used for family members will help to reduce population-wide salt intake. For salt reduction when dining out, more effective solutions may be led by the government which can learn from the many successful models that have been established around the world [23,24,25,26]. These models include developing an added salt standard for China’s catering industry and a clear labeling system for all processed foods and meals in order to help the public make healthy decisions. This would not only guide people to choose proper food and dining places outside their homes, but would also provide make more accessible foods healthier.

There are several limitations to this study. First, we did not use the 24-h urine collection method to measure salt intake for each individuals although we did a pilot study [8] comparing the two methods in parallel in 26 individuals from 11 families. The pilot work achieved a Pearson correlation coefficient of 0.762 between the “one-week salt estimation method” and 24-h urine collection, which is much higher than the correlation coefficient of 0.42 between 24-h dietary recall and 24-h urine collection [27,28]. The one-week salt estimation method we used detected a 1.8 g/person/day lower salt intake with comparison to the 24-h urine collection method. Although we had adjusted the amount of dietary salt intake in the current study based on the pilot study, the sample size of the pilot study is small and the exact value of underestimation may not be stable. Additionally, 10%–12% of salt intake derived from sodium is inherent to natural food [29], the method used only estimates salt intake from cooking or processing, did not take into consideration the sodium inherent to natural food. The adjusted amount of dietary salt intake in our study might not be overestimated. A larger study needs to be done to improve and validate the accuracy of the one-week salt estimation method in various populations. Second, we assumed that all the salt consumed dining out was from cooking which strictly speaking was not accurate. Most of the salt consumed at cafeterias or restaurants was added during cooking with very little of the salt was from processed food. To our knowledge, no study has shown the exact proportion of processed food consumed when dining out, but based on the food culture in China, we estimate this number is small. Therefore the results of our study which show 93.1% of the salt comes from cooking is slightly overestimated but still acceptable. Despite this second limitation, it is clear that participants consume far too much salt. The third limitation in this study was the representation of the samples. Because of various factors, we used convenience sampling which does not systematically represent the whole population. Therefore, the results should be used with caution in application and extrapolation.

## 5. Conclusions

In conclusion, our study has shown that the dietary salt intake in Beijing far surpasses the recommended amount by WHO. Cooking, including household, cafeteria and restaurant cooking, is still the major source of salt in the diet making up around 90% of total salt intake. More targeted interventions aimed both at altering domestic cooking habits and commercial cooking methods to use less salt should be undertaken to reduce the harm and risk posed by a high salt diet.
